# The effects of cognitive information processing and social cognitive career group counseling on high school students’ career adaptability

**DOI:** 10.3389/fpsyg.2022.990332

**Published:** 2022-09-02

**Authors:** Danqi Wang, Xiping Liu

**Affiliations:** Faculty of Psychology, Tianjin Normal University, Tianjin, China

**Keywords:** high school students, career intervention, CIP theory, SCCT theory, career adaptability

## Abstract

The aim of this study was to examine the effect of cognitive information processing (CIP) and social cognitive career theory (SCCT) group counseling on high school students’ career adaptability. The study involved 81 students from grade 10 and grade 11 in a Chinese public high school. Among the 81 participants, 27 were in the CIP group, 28 were in the SCCT group, while the rest were in the control group. All participants completed a pre-test, post-test, and tracking-test assessment of their career adaptability. Results indicated that the interventions were effective for the students with low career adaptability, the CIP group counseling improved the career concern after the intervention, whereas the SCCT group revealed a more robust effect on career adaptability after 3 months of the intervention. The practical implications of the study for career interventions are also discussed.

## Introduction

With the development of information technology and the economy, career patterns have become unpredictable at the present moment. Postmodern career systems are characterized by complexity and unpredictability (Bright and Pryor, 2005). Employment pressure and the outbreak of the COVID-19 resulted in a more severe employment situation. On the other hand, the Chinese university entrance examination has undergone significant changes in recent years. The new system’s impact on high school students is mainly reflected in the adjustment of examination time, the number of times, and examination subjects. These changes indicated that professional career counseling is urgently needed in China. The higher the career adaptability, the more flexible to cope with changing circumstances (Savickas and Porfeli, 2012). Therefore, there is urgent to enhance high school students’ career adaptability to cope with the changing external environment.

## Literature review

### Career adaptability

Career adaptability is described as “a psychosocial construct that denotes an individual’s resources for coping with current and imminent vocational development tasks, transitions, traumas” (Savickas and Porfeli, 2012, p. 662). Career adaptability consists of four dimensions: career concern, career control, career curiosity, and career confidence. Career concern essentially means focusing on future directions, preparing for the future, and feeling the future authentically. Career control indicates when individuals believe they are responsible for constructing their careers. Career curiosity involves the exploration of the relationship between oneself and the world of work. Career confidence refers to self-efficacy concerning one’s ability to construct their future and overcome possible difficulties (Savickas, 2013). These dimensions correspond to the four career questions “Do I have a future” “Who owns my future” “What do I want to do with my future” and “Can I do it” (Savickas, 2013). Career adaptability not only enhances high school students’ academic performance (Negru-Subtirica and Pop, 2016), but also predicts their life satisfaction (Marcionetti and Rossier, 2019). Therefore, career adaptability counseling is essential for high school students to help them adjust to school life and future development.

### Career intervention

At the moment, school career group counseling can be summarized in two main ways: one is based on cognitive information processing (CIP) theory, which means matching oneself to the professional world; the other is based on social cognitive career (SCCT) theory, which emphasizes a developmental perspective on individual goal selection based on previous learning experiences.

#### Cognitive information processing theory

Based on the process of information processing, researchers have constructed a “pyramid model” of information processing. The “pyramid model” has three components: the first level is the knowledge domain, including self-knowledge and occupational knowledge. Self-knowledge means learning oneself, including values, interests, and abilities. Occupational knowledge means knowing about careers, universities, and professions. The middle layer of the model is the decision-making skills domain, which consists of the CASVE cycle. The cycle consists of communication, analysis, synthesis, valuing, and execution. It refers to five steps in making good decisions. At the top of the model is the meta-cognitive domain, in which individuals think about the decision-making process. It tells the various programs at the second level how to operate. Meta-cognition is actually the perception of cognition, a higher level of cognition. There are three skills in the meta-cognitive domain: self-talk, self-awareness, control, and monitor (Sampson et al., 1992). The CIP model can be operationalized through career problem-solving which has an established record of successful career and employment services in over 40 developing countries with resource limitations (Toh and Sampson, 2019).

Career course based on CIP theory is an effective intervention method, which positively affected students’ career decision-making state, cognitive information processing skills, career knowledge, and anxiety about career concern (Osborn et al., 2020), increasing the graduation rate of college students (Reardon et al., 2015), rising in career certainty, satisfaction and clarity (Miller et al., 2018), improving self-efficacy and reduced difficulty during the major decision-making process for high school students (Dou et al., 2016). Researchers designed a CIP career intervention online during the COVID-19 pandemic, which demonstrated that the intervention significantly increased career readiness and reduced career decision-making difficulties among high school students (Chen et al., 2022). However, Bal and Arikan (2020) found the contrary statement. The group consisted of 41 undergraduate students in the psychology department of a university in Istanbul. The career course was prepared and conducted by the researcher during the fall term of 2019. The intervention lasted for 12 weeks, during which participants were involved in the themes as career planning introduction, knowing about myself and my options, and Career Decision-Making. The contrary result may be because the sample is undergraduates with professional tendencies. They have planned their career before choosing psychology. Therefore, CIP-based career counseling did not have an impact on their career adaptability.

#### Social cognitive career theory

Social cognitive career theory is based on Bandura’s social learning theory, which mainly includes self-efficacy, outcome expectations, and personal goals. The theory assumes that an individual’s self-efficacy, as well as outcome expectations, greatly impacts his interests, which in turn influence career choices and determine achievement performance (Lent and Brown, 1996). An individual’s self-efficacy and outcome expectations are derived from learning experiences. Learning experience is a crucial factor in promoting the development of career interests and the fundamental motivation of career interest development. A new study shows that experience, alternative learning, and positive emotions have different effects on self-efficacy. Among them, the effect of existing experience is the largest, followed by positive emotion and alternative learning. Verbal persuasion and negative emotion will not improve self-efficacy (Lent et al., 2017).

Social cognitive career theory has been successfully applied to a wide range of career services. A SCCT-based career counseling was designed, which positively affected participants’ career self-efficacy by four dimensions: achievement experience, alternative experience, verbal persuasion, and emotional response (O’Brien and Heppner, 1996). Betz and Schifano (2000) evaluated the effectiveness of career intervention to increase Realistic self-efficacy, such as using tools, assembling, building, and operating machines for female students. SCCT career group training included knowing about achievement, learning to search for career information, understanding interests and abilities, setting career goals, planning a career, overcoming difficulties, and taking job applications. It was conducted for undergraduates, which positively affected students’ career decision-making self-efficacy (Wang et al., 2010). Dantas and Azzi (2018) described the potential applications of the SCCT approach for a significant positive correlation between career self-efficacy and career interests of public high school students. In addition, researchers selected rural high school students as research participants for SCCT training, which showed that SCCT training can effectively improve students’ career exploration and planning for their future (Gibbons et al., 2019).

Although there has been much research in the field of career interventions (Xie and Long, 2007; Gu et al., 2020), there are relatively few research and practice programs on the effectiveness of interventions to enhance high school students’ career adaptability in China (Deng et al., 2020). Freshmen generally have low satisfaction with their majors, which may be because Chinese high school students generally find it hard to choose majors, and they lack knowledge and skills related to decision-making (Dou et al., 2016). The knowledge, decision-making, and metacognition of CIP are just what high school students lack. SCCT views personal career development in a developmental way. This theory emphasizes that goal selection is a dynamic process, which fits the characteristics of the current boundaryless era. Therefore, in order to enhance the career adaptability of high school students, this study explores CIP and SCCT intervention models for their ability to promote career adaptability under the perspective of career theory and prepare an intervention program based on the validation of its two intervention models.

## Materials and methods

### Purpose of the study

Due to the reform of the university entrance examination, high school students urgently need career counseling. Chinese high school students often have limited knowledge of themselves and the outside world, and hesitate to choose their majors (Chen et al., 2022), especially after the reform of the college entrance examination. SCCT intervention is effective for some high school students with developmental disabilities in rural areas (Gibbons et al., 2019). Therefore, CIP and SCCT may be more suitable for students with developmental disabilities, such as those with career adaptability at risk.

This study aimed to find out whether the CIP group or the SCCT group counseling had a significant effect on the career adaptability of high school students. In the present study, we made two main hypotheses: (1) Hypothesis 1: After the group counseling, the career adaptability of the CIP group will be higher than those of the control group. (2) Hypothesis 2: After the group counseling, the career adaptability of the SCCT group will be higher than those of the control group.

### Participants

Participants were selected from a public high school in Liaoning Province, China. Liaoning Province which is located in the northeast of China began to reform the college entrance examination in 2019. The reform requires students to select one subject from physics and history, and then select two subjects from chemistry, biology, politics, and geography. The educational resources in this area are relatively rare, and students have never been guided on career planning before, so many students will have problems after entering high school. This region is developing career planning courses to enable students to adapt to changes and improve their adaptability to the new college entrance examination.

First, high school students were measured using the Career Adaptability Scale, and then classroom presentations were used to recruit students based on convenience sampling. Inclusion criteria: (1) in the 27% bottom of career adaptability score; (2) interest in career planning and able to complete eight counseling sessions; (3) exclude those taking antidepressants within the last year. A total of 60 students entered formal counseling, and 55 students completed the whole intervention process. The purpose of the counseling was explained to the participants and informed consent was obtained. Our respective rights in the intervention were also clarified. Finally, following standard procedures, all participants signed an informed consent form.

Sample size estimation was performed using G*Power 3.1 software, based on repeated measures data ANOVA. Due to less research on career adaptability group counseling, the effect size was set to be moderate 0.25, the test level (*α*) was 0.05, the statistical test power (1–*β*) was 0.9, the number of groups was 3, the number of measurements was 3, and correlation coefficient for repeated measures was 0. The preliminary estimate of the total sample size required was 72.

Overall, 81 students (Girls = 43, 53.1%, Boys = 38, 46.9%) with an mean age of 15.46 years old (SD = 0.67) participated. The CIP group consisted of 27 participants (*M*_age_ = 15.41 years, SD = 0.69; Girls = 16, 59.3%, Boys = 11, 40.7%), with 28 participants in the SCCT group (*M*_age_ = 15.54 years, SD = 0.64; Girls = 14, 50%, Boys = 14, 50%), with 26 participants in the control group (*M*_age_ = 15.42 years, SD = 0.70; Girls = 13, 50%, Boys = 13, 50%).

### Measures

#### Demographic variables

A demographic questionnaire was asked to the participants as demographic items. These were presented in the last survey as grade, age, gender, birthplace, and parents’ educational levels, respectively.

#### Career adaptability

We used the Career Adapt-Abilities Scale-China Form (CAAS-*CF*; Hou et al., 2012) to measure participants’ career adaptability. The CAAS-*CF* contains 24 items: six items of career concern, career control, career curiosity, and career confidence, respectively. In addition, it is a five-point Likert scale in which the higher scores indicated more career adaptability. For the current study, the alpha coefficient of the total scale was 0.91. The four sub-scales registered internal consistencies of 0.84 (Career Concern), 0.82 (Career Control), 0.78 (Career Curiosity), and 0.85 (Career Confidence). The CFA revealed the four-factor model provided an acceptable fit to the data (*χ*^2^*/df* = 4.963, GFI = 0. 882, NFI = 0. 853, CFI = 0. 878, IFI = 0. 879, TLI = 0. 863, RMSEA = 0. 069).

#### Group member feedback form

The feedback form for group members was mainly used to give feedback on the immediate effect of members in the career counseling group. It includes the experience, gain, and suggestions about participating in the group.

## Design

Experimental group 1 was group counseling on cognitive information processing theory; experimental group 2 was group counseling on social cognitive career theory; while the control group attended self-study classes in class and did not receive any career-related counseling during the intervention period.

A mixed experimental design of 3 (groups: CIP group, SCCT group, and control group) × 3 (testing time: pre-test, post-test, and tracking test) was used to examine the effects of CIP and SCCT group counseling on high school students’ career adaptability. According to the time setting of group counseling and the students’ curriculum, group counseling was 1 h long each time and was delivered on a weekly basis.

Both the two experimental and the control group were administered the Career Adapt-Abilities Scale as a post-test and tracking test. In addition, an open-ended questionnaire was sent to the students in experimental group 1 and group 2 after the last session, which mainly asked them to write about the biggest changes from attending the counseling and suggestions for the course.

## Procedure

Students participated in two types of career interventions: CIP group and SCCT group. The two group interventions were administered by an experienced school psychology teacher.

The program for the CIP group consisted of a “pyramid model” of cognitive information processing, organized into three modules: Module 1: knowledge (self-knowledge, occupational knowledge), Module 2: the CASVE cycle of decision-making (communication, analysis, synthesis, valuation, and execution), Module 3: meta-cognition. In terms of practical basis, the meta-cognitive career counseling is based on interviews with some students, and the program uses strategies for developing self-control and problem solving (Hou, 2016).

CIP group was composed of eight sessions: (1) Starting group: This section aimed to form groups, understand group goals, and development of group norms. (2) Self-knowledge and occupational knowledge: The second session aimed to increase career self-knowledge and occupational knowledge by measuring Multiple Intelligence before group counseling, realizing superior intelligence, and knowing the major that corresponds to their superior intelligence. (3) Communication: We highlighted the importance of selecting their majors in career development. Then, we discussed and shared ways how to select a major. (4) Analysis: This is the stage of understanding myself and my choices. Students were asked to identify the three majors that best matched their interests and abilities through Holland Vocational Interest Island activity and the previous multiple intelligence. (5) Synthesis and Valuation: First, we introduced students to the occupational and major classifications. Students were encouraged to identify the 3–5 majors they wanted to choose. Second, students were guided in filling out the career decision balance sheet and ranking their major categories. (6) Execution: We led students to realize that they need to study hard and improve their academic performance to achieve their goals. Finally, we discussed and shared learning strategies. (7) Meta-cognitive: We introduced self-control strategies through panel discussions and guided students to find solutions to problems through drawing. (8) Message on back: We reviewed the process of group activities and left messages of blessing to each other.

The SCCT-oriented group counseling was based on social cognitive career theory, and the program included learning experiences, career self-efficacy, and outcome expectations. In terms of practical basis, the SCCT-oriented group counseling mainly refers to the activity design of Jin (2007), including lifelines, a letter to the future, and a life fantasy tour.

SCCT group was also composed of eight sessions: (1) Starting group: This section aimed to the formation of groups, understand group goals, and development of group norms. (2) My achievement story: The members’ were focused on recalling past events of achievement, and they became aware of what strengths they have. (3) Lifelines: We guided participants to depict a lifeline with significant life events, which prompted them to share and reflect on their personal life stories of past events, present challenges, and future projects. (4) Life drawing book: The members were invited to draw their lives pictorially by making drawings. Finally, a small book is formed about their past, present, and future. (5) Life fantasy tour: This section aimed to assist members in constructing a vision of their future career by asking them to imagine a typical day after 10 years. (6) Future business card: The participants were asked to design their future business cards in the workplace, including job title, company name, location, nature of the company, and future life they want to lead. Then, they discussed a series of questions and thought about what could be done to work toward the goal of the future business card. (7) A letter to the future: We guided members to think about what kind of person they want to be and write a letter to the future themselves. (8) Message on back: We reviewed the process of group activities and left messages of blessing to each other.

## Data analysis

All analyses were conducted by using SPSS 25.0. To evaluate the effectiveness of career interventions on three groups of students, the analyses were carried out in two steps: First, One-way ANOVA was tested to compare the differences at baseline levels on career adaptability and four dimensions. Second, a repeated-measures ANOVA was conducted to examine the interaction effect between group (CIP group vs. SCCT group vs. Control group) and time (pre-test vs. post-test vs. tracing-test) in career adaptability and four dimensions. Lachenbruch and Cohen’s (1989) *η*^2^*_p_* were calculated to assess the percentage of variance explained by the variable. Conventionally, the threshold values for the index *η*^2^*_p_* are 0.01, 0.06, and 0.14, which represent a small, moderate, and large effect size, respectively.

## Results

### Pre-test data analysis

A one-way ANOVA was used to examine the participants before the intervention in career adaptability and its four dimensions. Table 1 presents the results of the one-way ANOVA of the pre-test among participants’ scores on Career Adaptability, Career Concern, Career Control, Career Curiosity, and Career Confidence. The results demonstrated that the differences in career adaptability and the four dimensions of the three groups were not significant (*p* > 0.05), which indicated that the groups were homogeneous before the intervention (Table 1).

**Table 1:** Pre-test of career adaptability scores among three groups.

	CIP group (*n* = 27)	SCCT group (*n* = 28)	Control group (*n* = 26)	*F*	*p*
Career concern	3.48 ± 0.69	3.88 ± 0.45	3.73 ± 0.61	0.699	0.500
Career control	3.70 ± 0.54	3.73 ± 0.86	3.68 ± 0.46	0.044	0.957
Career curiosity	3.41 ± 0.72	3.47 ± 0.68	3.38 ± 0.69	0.120	0.887
Career confidence	3.17 ± 0.67	3.29 ± 0.54	3.09 ± 0.55	0.814	0.447
Career adaptability	3.44 ± 0.50	3.46 ± 0.38	3.43 ± 0.29	0.053	0.948

### Effects of different interventions on career adaptability and the dimensions

Supplementary Figure 1 presents the results of repeated measures with the change in career adaptability. A repeated-measures ANOVA reveals a significant group × time interaction [*F*_(4, 156)_ = 6.253, *p* < 0.001, Partial *η*^2^ = 0.138]. Further simple effect analysis reveals significant differences between social cognitive career theory (SCCT) and control group at T2 and T3 (*p* < 0.001), the same as the SCCT and cognitive information processing (CIP) groups (*p* < 0.01). However, career adaptability in the SCCT group was significantly higher in T2 and T3 than T1 (*p* < 0.001), and no significant differences between the other groups at each time point (*p* > 0.05).

Supplementary Figure 2 presents the results of repeated measures with the change of career concern. For career concern, the group × time interaction was significant [*F*_(4, 156)_ = 4.311, *p* < 0.01, Partial *η*^2^ = 0.100]. Further simple effect analysis revealed significant differences between the SCCT and control groups at T2 and T3 (*p* < 0.05), and significant differences between CIP and control groups at T2 (*p* < 0.05). Career concern was significantly higher with the SCCT group in T2 and T3 than T1 (*p* < 0.001), CIP group was significantly higher in T2 than T1(*p* < 0.01). However, there was no significant difference between the other groups at each time point (*p* > 0.05).

Supplementary Figure 3 presents the results of repeated measures with the change of career control. For career control, the group × time interaction was not significant and the main effect of group was marginal significance [*F*_(2, 78)_ = 2.564, *p* = 0.083, Partial *η*^2^ = 0.062]. Further simple effect analysis reveals significant differences between the SCCT and control groups at T2 and T3 (*p* < 0.05). Career control in the SCCT group was significantly higher in T3 than T1 (*p* < 0.05). There was no significant difference between the other groups at each time point (*p* > 0.05).

Supplementary Figure 4 presents the results of repeated measures with the change of career curiosity. For career curiosity, the group × time interaction was significant [*F*_(4, 156)_ = 2.851, *p* < 0.05, Partial *η*^2^ = 0.068]. Further simple effect analysis revealed significant differences between the SCCT and control groups at T2 and T3 (T2, *p* < 0.01; T3, *p* < 0.05), and between the SCCT and CIP groups at T2 (*p* < 0.001). Career curiosity in the SCCT group was significantly higher in T2 and T3 than T1 (T2, *p* < 0.01; T3, *p* < 0.05). There was no significant difference between the other groups at each time point (*p* > 0.05).

Supplementary Figure 5 presents the results of repeated measures with the change in career confidence. For career confidence, the group × time interaction was significant [*F*(4, 156) = 4.239, *p* < 0.01, Partial *η*^2^ = 0.098]. Further simple effect analysis reveals significant differences between the SCCT and control groups at T2 and T3 (*p* < 0.001), and between the SCCT and CIP groups at T2 and T3 (*p* < 0.001). Career confidence in the SCCT group was significantly higher in T2 and T3 than T1 (*p* < 0.001), with no significant differences between the other groups at each time point (*p* > 0.05).

### Qualitative data analyses

After the group activities, most of the students in the experimental group said that they “have more goals now,” “can examine themselves more deeply and know what they want more,” “are more firm in their goals” and “their goals are clearer and more motivated.” Another part of the students wrote that “they have a better understanding of themselves and know what they are suitable for” and “they have a deeper understanding of the majors they want to choose.” Some students mentioned that “I am more confident now” and “I have made some new friends and I am more cheerful than before.”

The analysis of the open-ended question of the CIP group counseling for the students yielded the following major themes: making a clear target, knowing major information, confidence for the future, being open and cheerful, thinking more about the future, knowing self-information, and increment confidence. Similarly, the analysis of the students’ open-ended question of the SCCT group counseling yielded the following major themes: making a clear target, increment confidence, making a firm target, being open and cheerful, and thinking more about the future.

## Discussion

Results indicated that the SCCT-based intervention increased high school students’ career adaptability, as predicted by Hypothesis 2. Unlike what was predicted by Hypothesis 1, CIP-based intervention did not improve high school students’ career adaptability.

Our study has described the CIP-based intervention was not significant in improving high school students’ career adaptability, which is consistent with previous research on college students (Bal and Arikan, 2020). Bal and Arikan (2020) used CIP theory as the basis for their intervention, which consisted of four sections: “Introduction to Career Planning,” “Knowing about Myself,” “Knowing about My Options,” and “Career Decision-Making.” The reason for the insignificant effect may be that the efficacy factor of the career adaptability intervention is meta-cognitive. Researchers used cognitive behavioral therapy counseling for nursing students of eight sessions and showed that the cognitive counseling increased the students’ career maturity (Lim et al., 2010). The CIP group counseling in our study, which was about self-knowledge, occupational knowledge, and how to make decisions, the only session about meta-cognition, maybe the main reason for the limited effect of the CIP group on career adaptability. On the other hand, it may be because high school students are still in the exploratory stage of their careers, and CIP emphasizes person-job matching where individuals are in a stable state.

The results of the CIP group showed that there was a transient increase in career concern. At the immediate end of the intervention, there was a significant difference in career concern compared to pre-intervention, which may be due to the self-knowledge and professional knowledge involved in the counseling. However, after 3 months, the career concern returned to the pre-test level, which may be due to the decrease in students’ willingness to explore career issues after group counseling. The effect of career concern was limited to the counseling and was not sustainable. As a result, high school students with low career adaptability are not impacted by the CIP-based intervention.

In another group, SCCT counseling significantly improved students’ career adaptability and had lasting intervention effects, which is consistent with the results of previous studies (Janeiro et al., 2014). Researchers compared the effects of career courses and group career counseling on career adaptability, which showed that career courses increased career curiosity and career confidence in only one group of students. In contrast, group career counseling had a more significant effect on students with insecure, pessimistic, or superficial career coping styles. What makes counseling effective can be explained by the effectiveness component of the career intervention (Whiston et al., 2017), which the meta-analysis found that effective career counseling interventions were namely written exercises for participants to clarify their goals and plans, individualized interpretation and feedback. The SCCT group’s outcome expectations correspond to this crucial component.

As seen from the effect sizes, the time and group interaction partial eta-squared for career concern, career curiosity, career confidence, and career adaptability was moderate or above. Only career control was lower, there was no significant difference in SCCT group after the intervention, but after 3 months of the intervention, there was a borderline significant. Career control was somewhat improved after 3 months, and this may be because students could still actively explore content related to career planning after the group counseling.

Unlike the person-job matching advocated by mainstream career counseling in the past, SCCT-based counseling approaches career planning from a developmental perspective, identifying interests, goals, and actions from learning experiences, career self-efficacy, and outcome expectations. By finding strengths from learning experiences, accepting oneself, and improving career self-efficacy, students can construct outcome expectations for their careers. In particular, “My Achievement Story” enables students to find their strengths, improve their self-efficacy, boost their confidence in themselves and their future development, and be better prepared for their future career development. “My Lifeline” and “Life Drawing Book” help students explore their own life journey and see life themes through reflection, based on which they can plan for their career, realize that all they can do is work toward their future goals, and only they can control their future themselves. The “Career Fantasy Tour,” “Future Business Card” and “Letter to the Future” helped members explore their desired vision of their future lives, further motivating them to imagine future scenarios. The scenario will focus members on what they want to do in the future of career curiosity, which triggers a focus on the future.

Mainstream career construction or life design requires in-depth interviews with participants, and the number of participants is limited. CIP focuses on career knowledge, decision-making, and metacognition; SCCT focuses on self-efficacy, outcome expectation, and learning experience. For group counseling of about 30 participants, CIP and SCCT may be more suitable. The intervention programs received by the participants in CIP and SCCT groups were designed on theoretical and practical bases. The CIP-based intervention was more systematic in the content system, with lecture-based courses a greater emphasis on career knowledge. The SCCT-based intervention was more engaging in content and easily triggered students’ interest, with less theoretical career knowledge and more focus on exploration, discussion, and sharing. Each of the two types of counseling has its own advantages, mainly in the systematization of the CIP, which can be carried out on a large scale of lectures. However, SCCT group requires a high level of tutors and has a limited number of members.

Self-compiled group member feedback complemented the quantitative study, and career counseling members of both orientations showed changes related to future planning, interpersonal communication, and confidence.

## Implications for practice

Career adaptability intervention program was set up at the practice level, which had a corresponding theoretical basis, compared two mainstream career counseling techniques, and provided a set of models for career interventions that will help provide an effective reference for career counseling in the future.

Our study demonstrated that interventions based on the CIP theory have a reduced impact on high school students with low career adaptability. Moreover, our findings showed that students’ career adaptability gets improved under SCCT, which testifies the efficacy of this career group counseling. The SCCT group counseling can assist students in developing a “positive but uncertain” view of their careers.

## Limitations and recommendations for future research

Although the present research has yielded several significant implications, it is important to recognize several limitations. First, the research participants are limited in the sampling and coverage as only students from a high school with low career adaptability are chosen as participants. Scholars in the future are expected to conduct empirical studies involving more participants at different levels so that the sample’s representativeness can be guaranteed. Second, relying only on the CAAS to measure counseling effect seemed insufficient. Future research could also consider the impact of the group intervention on other variables linked to career counseling, such as career decision-making self-efficacy. In addition, future research will consider optimizing the counseling program and enhancing the promotional of career counseling. According to different needs, schools can combine both CIP and SCCT programs.

## Data availability statement

The original contributions presented in the study are included in the article/Supplementary material, further inquiries can be directed to the corresponding author.

## Ethics statement

Ethical review and approval was not required for the study on human participants in accordance with the local legislation and institutional requirements. Written informed consent to participate in this study was provided by the participants' legal guardian/next of kin.

## Author contributions

DW and XL contributed to design of the study. DW wrote the manuscript. XL modified the manuscript. All authors contributed to the article and approved the submitted version.

## Conflict of interest

The authors declare that the research was conducted in the absence of any commercial or financial relationships that could be construed as a potential conflict of interest.

## Publisher’s note

All claims expressed in this article are solely those of the authors and do not necessarily represent those of their affiliated organizations, or those of the publisher, the editors and the reviewers. Any product that may be evaluated in this article, or claim that may be made by its manufacturer, is not guaranteed or endorsed by the publisher.
