# Supplementary material for: The effects of cognitive information processing and social cognitive career group counseling on high school students’ career adaptability
Source: Front Psychol. 2022 Sep 2;13:990332. doi: 10.3389/fpsyg.2022.990332 (PMC9480515; doi:10.3389/fpsyg.2022.990332)
Supplement: Supplementary file 1 [file Data_Sheet_1.PDF]

Supplementary Material

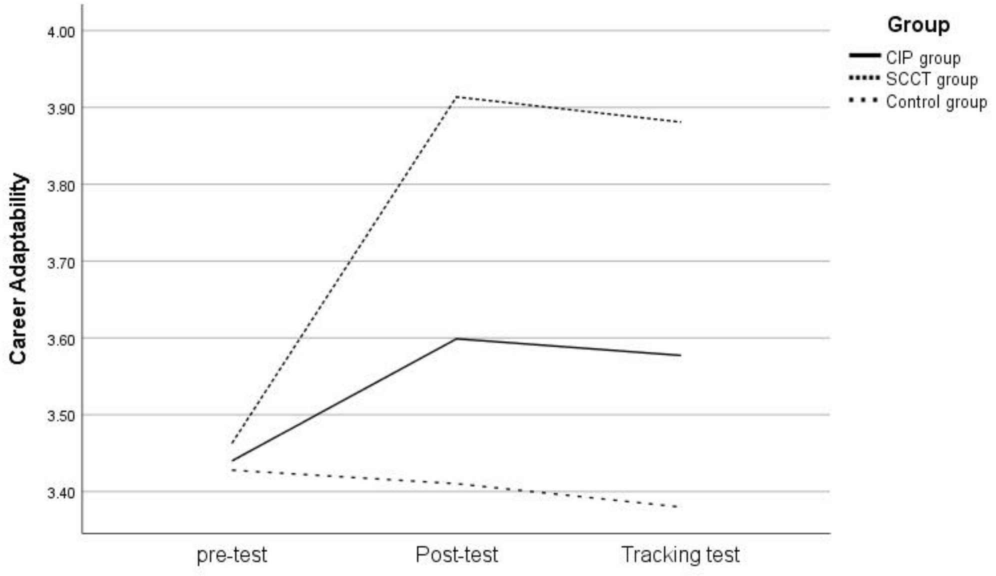

Figure 1 Career adaptability in three groups at three time points

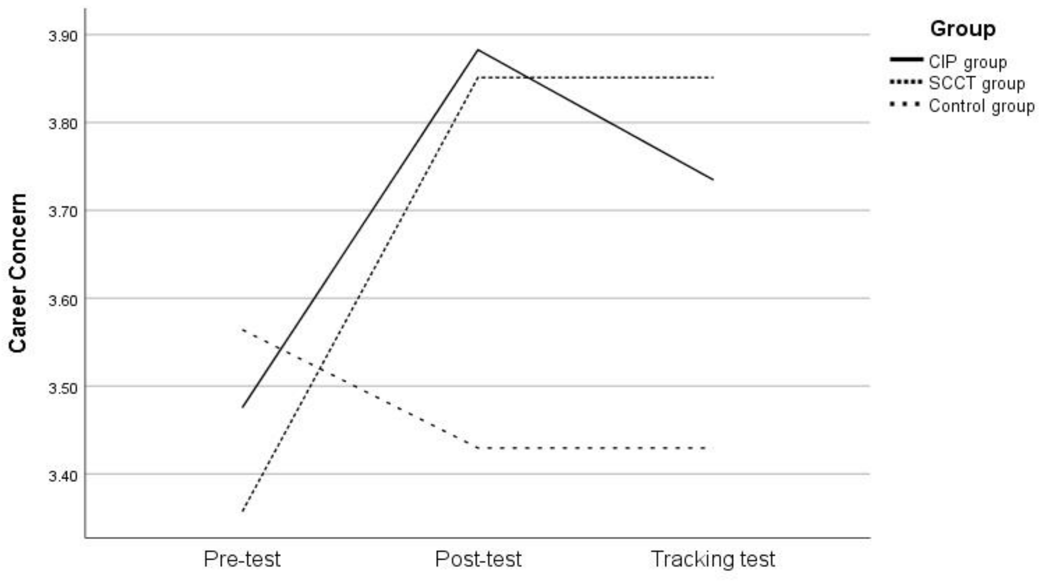

Figure 2 Career concern in three groups at three time points

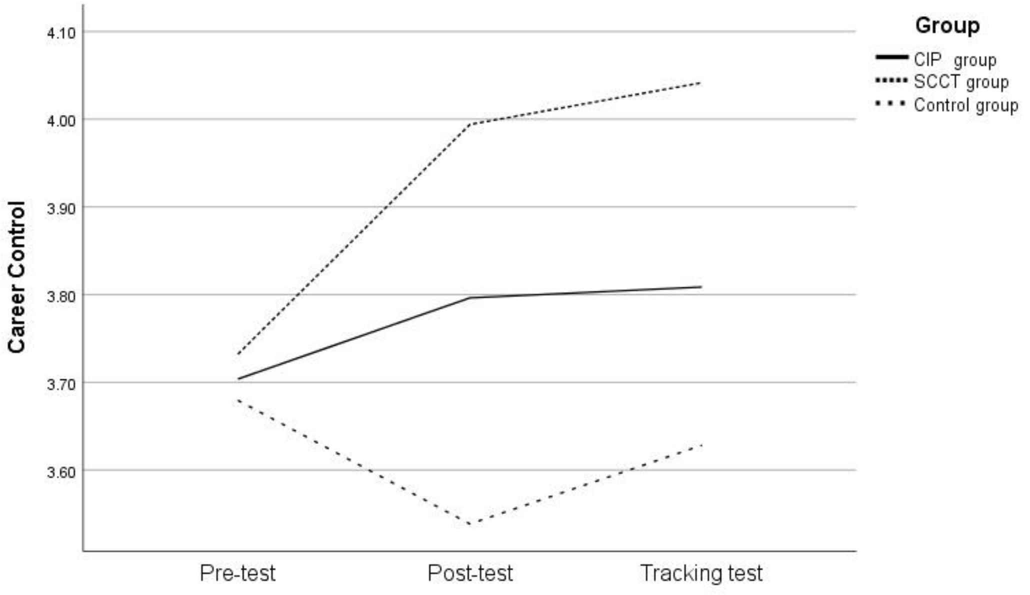

Figure 3 Career control in three groups at three time points

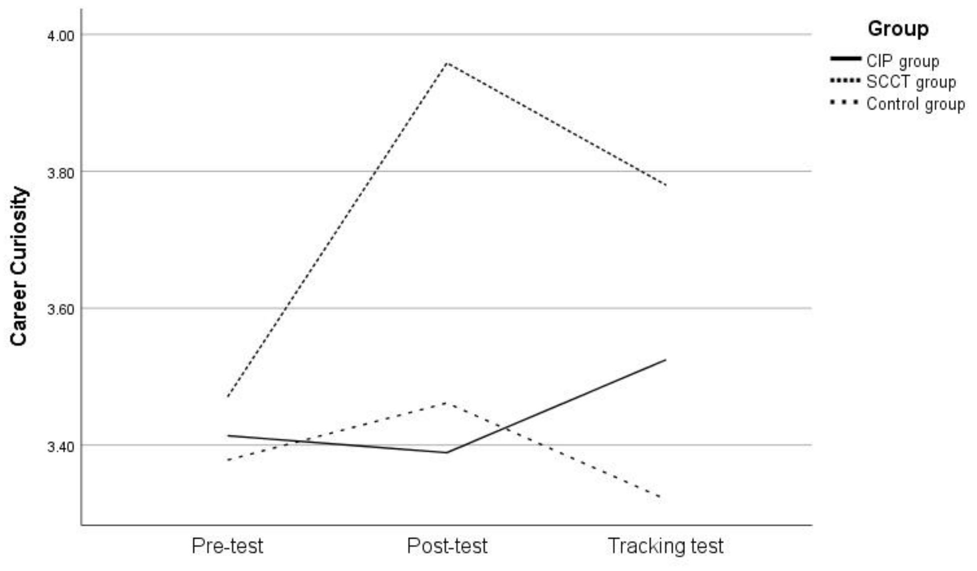

Figure 4 Career curiosity in three groups at three time points

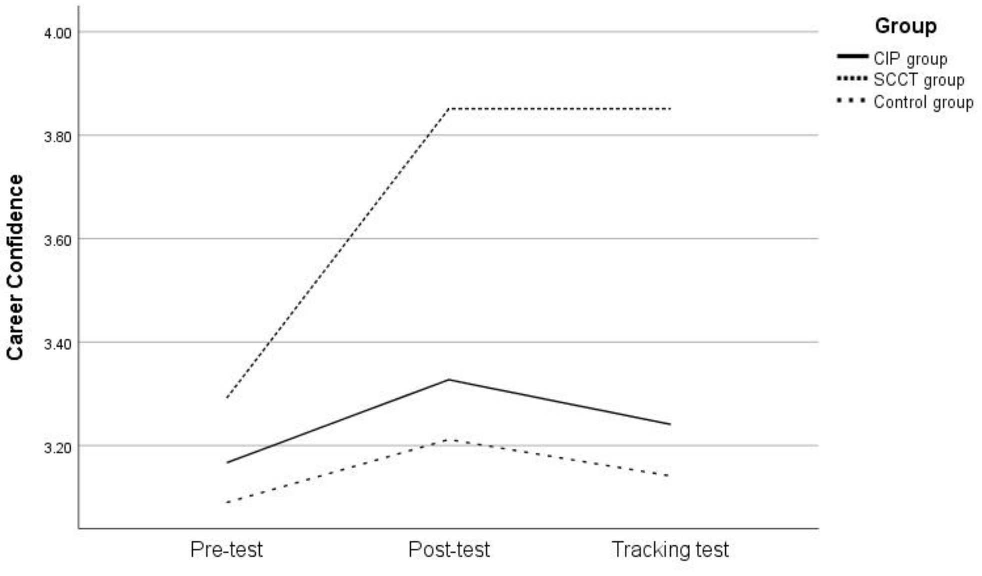

Figure 5 Career confidence in three groups at three time points
